# Ruling out pulmonary embolism across different subgroups of patients and healthcare settings: protocol for a systematic review and individual patient data meta-analysis (IPDMA)

**DOI:** 10.1186/s41512-018-0032-7

**Published:** 2018-07-02

**Authors:** G.-J. Geersing, N. Kraaijpoel, H. R. Büller, S. van Doorn, N. van Es, G. Le Gal, M. V. Huisman, C. Kearon, J. A. Kline, K. G. M. Moons, M. Miniati, M. Righini, P.-M. Roy, S. J. van der Wall, P. S. Wells, F. A. Klok

**Affiliations:** 1 Julius Center for Health Sciences and Primary Care, University Medical Center Utrecht, Utrecht University, Heidelberglaan 100, 3584 CX Utrecht, the Netherlands; 2 Academic Medical Center, Vascular Medicine, University of Amsterdam, Amsterdam, the Netherlands; 3 Department of Medicine, University of Ottawa, Ottawa Hospital Research Institute, Thrombosis Research Group, Ottawa, Canada; 4 Department of Thrombosis and Hemostasis, Leiden University Medical Center, Leiden University, Leiden, the Netherlands; 5 Department of Medicine, The Thrombosis and Atherosclerosis Research Institute, Mc Master University, Hamilton, Canada; 6 School of Medicine, Indiana University, Indianapolis, IN USA; 7 Department of Medicine, University of Florence, Florence, Italy; 8 Division of Angiology and Haemostasis, Geneva University Hospitals and Faculty of Medicine, Geneva, Switzerland; 9 Emergency Department, University of Angers, Angers, France

**Keywords:** Pulmonary embolism, Clinical prediction model, D-dimer, Individual patient data meta-analysis

## Abstract

**Background:**

Diagnosing pulmonary embolism in suspected patients is notoriously difficult as signs and symptoms are non-specific. Different diagnostic strategies have been developed, usually combining clinical probability assessment with D-dimer testing. However, their predictive performance differs across different healthcare settings, patient subgroups, and clinical presentation, which are currently not accounted for in the available diagnostic approaches.

**Methods:**

This is a protocol for a large diagnostic individual patient data meta-analysis (IPDMA) of currently available diagnostic studies in the field of pulmonary embolism. We searched MEDLINE (search date January 1, 1995, till August 25, 2016) to retrieve all primary diagnostic studies that had evaluated diagnostic strategies for pulmonary embolism. Two authors independently screened titles, abstracts, and subsequently full-text articles for eligibility from 3145 individual studies. A total of 40 studies were deemed eligible for inclusion into our IPDMA set, and principal investigators from these studies were invited to participate in a meeting at the 2017 conference from the International Society on Thrombosis and Haemostasis. All authors agreed on data sharing and participation into this project. The process of data collection of available datasets as well as potential identification of additional new datasets based upon personal contacts and an updated search will be finalized early 2018. The aim is to evaluate diagnostic strategies across three research domains: (i) the optimal diagnostic approach for different healthcare settings, (ii) influence of comorbidity on the predictive performance of each diagnostic strategy, and (iii) optimize and tailor the efficiency and safety of ruling out PE across a broad spectrum of patients with a new, patient-tailored clinical decision model that combines clinical items with quantitative D-dimer testing.

**Discussion:**

This pre-planned individual patient data meta-analysis aims to contribute in resolving remaining diagnostic challenges of time-efficient diagnosis of pulmonary embolism by tailoring available diagnostic strategies for different healthcare settings and comorbidity.

**Systematic review registration:**

Prospero trial registration: ID 89366.

## Background

Signs and symptoms of acute pulmonary embolism (PE) are non-specific and may range from coughing, shortness of breath, chest pain, or syncope. Given its potential severity, physicians have a low threshold for referring a patient at suspicion of PE for diagnostic imaging. As a result, the proportion of confirmed cases among those with suspected PE is low and has decreased over recent years [1]. Some authors have stressed that the threshold for referral for computed tomography pulmonary angiography (CTPA; reference standard for PE) is too low, risking contrast nephropathy and radiation-induced cancer in too many patients [2]. In addition, CTPA may detect small sub-segmental emboli, of which the clinical relevance remains unclear but are nonetheless often treated with anticoagulants, which confers a bleeding risk [3]. Rapid and accurate selection of those patients requiring CTPA is therefore of paramount importance. The current recommended diagnostic approach starts with clinical pre-test probability assessment using a validated clinical decision rule (CDR). In those with low pre-test probability, negative D-dimer testing can safely reduce the number of referrals for imaging in about 30% of suspected patients [4].

Nevertheless, it is increasingly recognized that CDRs and D-dimer testing may not be as effective and safe for all subgroups of patients. Importantly, D-dimer testing has a low specificity, meaning that the test often yields false-positive results, especially in elderly patients with comorbidity, cancer patients, and hospitalized patients, but also in younger patients with a (very) low clinical probability, or those with a history of PE [5–7]. To increase the specificity of D-dimer testing, it has been suggested to adapt the interpretation of the D-dimer result using a threshold adjusted to age or clinical pre-test probability [8].

Second, important differences in case-mix and healthcare settings (e.g., emergency ward, primary care, secondary care, or nursing home) exist. It has previously been demonstrated that these differences relate to PE prevalence in the suspected population, which influences the predictive performance of CDRs [4]. As a consequence, several CDRs have been developed and validated, which all have specific advantages and limitations. Typically, these CDRs are only validated *within* the setting in which they have been developed, yet validation studies *across* different healthcare settings (all with a different PE prevalence) or subgroups are limited or non-existing altogether.

Despite advances made, these issues leave clinicians with uncertainty about the appropriate diagnostic approach for a patient in a specific healthcare setting. Rather than performing a new prospective study addressing these issues, an alternative and much more convenient, less costly, and faster novel approach is to combine individual patient data (IPD) from existing studies. IPD meta-analysis is a powerful method that allows for robust model validation and updating techniques across multiple healthcare settings and subgroups [9, 10]. We recently performed such an IPD meta-analysis (IPDMA) for diagnosing deep vein thrombosis (DVT) and for evaluation of the validity of CDRs and D-dimer testing in a selected secondary care (referred) population with suspected PE [6, 11]. This paper describes the protocol of a large international IPD meta-analysis for ruling-out PE across different subgroups and healthcare settings.

## Methods/design

This IPDMA will follow the guidance of *Preferred Reporting Items for Systematic reviews and Meta-Analyses of Individual Participant Data (PRISMA-IPD) Statement* [12]. For this protocol paper, we adhere to the guideline for Preferred Reporting items for Systematic Review and Meta-Analysis Protocols (PRISMA-P) Statement, explained in Appendix [13].

### Study eligibility criteria

Eligible studies are those that (1) have a prospective or cross-sectional design, including patients with clinically suspected PE, (2) report original data on (the method of) pre-test probability assessment and assess variables to calculate at least one prediction rule; studies only evaluating “gestalt” or an implicit pre-test probability assessment, thus without the use of an objective prediction rule, will be excluded, (3) include a clear description of the source of patient enrolment or clinical healthcare setting; studies only including children or pregnant women are not eligible for inclusion, (4) have objectively confirmed PE diagnosis with either imaging (CTPA, ventilation-perfusion lung scan or digital subtraction angiography) or clinical follow-up of at least 1 month in those initially not having received anticoagulant treatment based on the initial diagnostic testing, and (5) have at least 50 patients with confirmed PE.

### Search strategy

A systematic search was conducted in MEDLINE from January 1, 1995, to August 25, 2016, using a previously developed search string for prediction development or validation studies in MEDLINE [14], combined with terms for pulmonary embolism (see the Appendix). This search string has a high sensitivity for retrieving studies developing or validating a (formal) clinical prediction models, such as a clinical pre-test probability assessment method for diagnosing PE (e.g., the Wells rule or the PERC model). No language restrictions were applied. Two reviewers (GJG and NK) independently screened titles and abstracts, and subsequently, four reviewers (GJG, NK, NvE, and FAK) independently assessed the full-text articles for eligibility. Disparate conclusions were resolved by discussion.

**Fig. 1:**
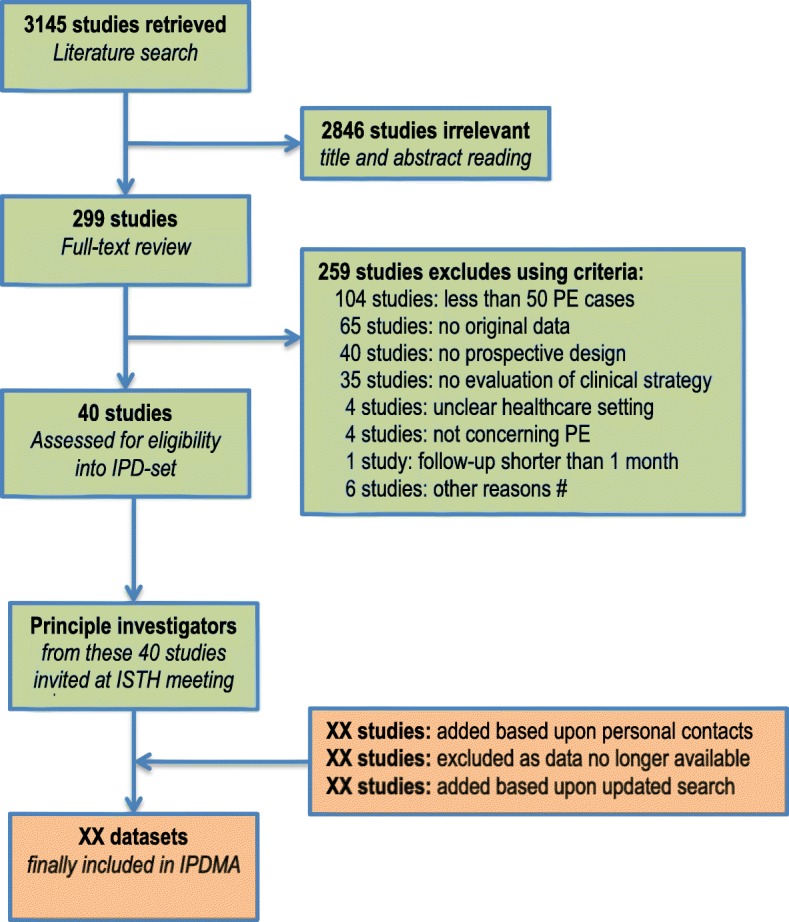
Flowchart of included studies

A total of 3145 individual studies were assessed for eligibility, leading to 40 potentially retrieved studies. The results of this literature search were discussed during a meeting at the International Society on Thrombosis and Haemostasis conference in Berlin (2017) with all principal investigators from these 40 retrieved studies. Each principal investigator checked the retrieved list of studies for completeness and data availability and was asked to suggest additional studies or sources not retrieved by the literature review, if these studies fulfilled our pre-defined eligibility criteria. This final stage of study identification and individual patient data collections is currently ongoing and will be completed early/mid 2018, including an update of our time limit (currently August 25, 2016) for the search to the most convenient recent date. Accordingly, a set of included studies with available datasets on an individual level will be constructed in order to build the final individual patient dataset, see Fig. 1 for the current flow of our search strategy.

The systematic review is registered in the PROSPERO database for systematic reviews (ID 89366).

### Handling of missing data

To avoid bias induced by ignoring missing data in clinical research, it is widely acknowledged that (multiple) imputation techniques should be considered to replace missing values. For this IPDMA, we consider imputation (particularly) appropriate in the context of the missing at random situation, where the reason for missing values is correlated with observed values in other patients. Data can be either partially or systematically missing in a dataset. For partially missing data, traditional multiple imputation techniques will be performed per individual dataset, if not yet done by the researchers from the respective paper (in which case this imputed dataset will be used instead) and also if the proportion of missing values in relation to the total dataset is reasonably small allowing for the construction of a robust imputation model. For completely or systematically missing data, more advanced methods for imputation will be performed where appropriate by using state-of-the-art statistical techniques, preferably imputing systematically missing data with partially missing data into one imputation model [15]. All statistical analyses described below will be performed only after imputation of missing values, yet we will describe the proportion of missing values for each dataset included in our IPDMA.

### Clinical decision rules under evaluation

The main advantage of clinical pre-test probability assessment is that it can be performed at the bedside. Thus, the CDR is based on readily available information on the patient’s medical history and physical examination, followed by D-dimer testing when needed. Recently, Hendriksen and colleagues performed a systematic literature review with the purpose of identifying available and easily applicable CDRs for suspected PE [16]. We aim to validate—and update if needed—these CDRs and add a novel CDR that was published after the aforementioned literature review (i.e., the YEARS rule) [17]. These clinical decision rules are summarized in Table 1.

**Table 1:** Clinical decision rules under evaluation

Wells’ score [21]		Revised Geneva [22]		PERC [23]	YEARS [24]
Items	CDR points	Items	CDR points	Items	Items
Previous VTE	1.5	Previous VTE	3	Age < 50 y	Clinical signs of DVT
Heart rate > 100/min	1.5	Heart rate 75–94/min ≥ 95/min	3 5	Pulse < 100/min	Hemoptysis
Surgery or immobilization < 4 weeks	1.5	Surgery or fracture < 1 month	2	Saturation > 94%	PE most likely diagnosis
Hemoptysis	1	Hemoptysis	2	No unilateral leg swelling	Clinical probability: 0 items and D-dimer < 1000 ng/ml: PE ruled out 0 items and D-dimer ≥ 1000 ng/ml: Likely ≥ 1 items and D-dimer < 500 ng/ml: PE ruled out ≥ 1 items and D-dimer ≥ 500 ng/ml: Likely
Active cancer	1	Active cancer	2	No hemoptysis	
Clinical signs of DVT	3	Unilateral lower limb pain	3	No recent trauma or surgery	
Alternative diagnosis less likely than PE	3	Pain on lower limb, deep venous palpation, and unilateral edema	4	No estrogen use	
Clinical probability: Low < 2 total Intermediate 2–6 total High > 6 total Unlikely ≤ 4 Likely > 4		Age > 65 years	1	No previous VTE	
		Clinical probability: Low 0–3 Intermediate 4–10 High ≥ 11		Clinical probability: PE ruled out when fulfilling these criteria	
Further testing: • “Unlikely,” “Low,” or “Intermediate” plus abnormal D-dimer testing, and • “High”, or “Likely”, regardless of D-dimer testing (i.e., D-dimer testing not indicated) In all other patients, PE is considered ruled-out.		Further testing: • “Low”, or “Intermediate” plus abnormal D-dimer testing. • “High”, regardless of D-dimer testing (i.e., D-dimer testing not indicated) In all other patients, PE is considered ruled-out.		Further testing: All patients fulfilling at least one PERC item. In all other patients, PE is considered ruled-out.	Further testing: All patients classified into “Likely”. In all other patients, PE is considered ruled-out.

### Statistical analyses and objectives addressed by this review

Although we acknowledge that with emerging knowledge and evidence, additional novel questions may arise that could be addressed within this IPDMA leading to potential amendments to this protocol. Should such amendments be necessary, these will be explained thoroughly in the respective future publications. Nevertheless, we now first aim to address the following three clinically important research domains related to the management of suspected PE:

#### Research domain 1: what is the optimal method for assessing clinical pre-test probability across different healthcare settings?

The optimal method of pre-test probability assessment is likely different across varying health settings due to intrinsic differences in patient characteristics and the prevalence of PE in the suspected population. For instance, in open-access emergency care, the PE prevalence is typically 5% or less compared to 20–30% for an in-hospital setting [4]. Whereas clinical decision rules with a moderate to high sensitivity but high specificity may be preferred in the first setting, only high sensitivity clinical decision rules with moderate specificity are acceptable in the second.

Studies will be categorized in the following healthcare settings, dependent on the overall PE prevalence as well as the clinical context in which the study is performed:

I. *Open-access emergency care*: this setting is defined by patients presenting themselves, typically without referral, to an emergency care department. The overall prevalence of confirmed PE is 5% or less.
II. *Primary healthcare*: in this setting, patients are seen on an outpatient clinic, usually by a general physician, family doctor or general internist, who needs to decide on the need for further referral, based on contextual knowledge, clinical pre-test probability assessment and D-dimer testing. The overall PE prevalence usually is 5–15%
III. *Emergency ward or hospital-care setting*: this setting differs from open-access emergency care by the fact that the target population is referred based upon a clear suspicion of acute PE, usually by a family doctor or general internist. The overall prevalence usually is 15–25%.
IV. *In-hospital or nursing home setting*: this setting covers both hospitalized in-patients with acute disease or after surgery, and old and frail institutionalized patients who are cared for in a long-term clinical setting. The overall prevalence of PE is typically high, e.g., > 25%.

In each setting, we aim to validate all clinical decision rules summarized in Table 1. Discrimination will be quantified using the concordance (c)-statistic and visualized by plotting a ROC curve. Calibration will be graphically illustrated in a calibration plot and quantified by assessing the calibration slope in this plot as well as calculate the expected versus observed ratio (with good calibration implying that both should be equal to, or at least approaching, 1). Finally, the diagnostic indices for each rule (sensitivity, specificity, predictive values) will be calculated using rule-specific thresholds (i.e., the distinction into “in need for further testing” versus “PE considered ruled-out”; see Table 1). The diagnostic indices that are usually reported for CDR studies investigating PE are the safety and efficiency. Safety is defined as the proportion of patients with a negative strategy (low score on CDR and normal D-dimer level) that are still diagnosed with venous thromboembolism (deep vein thrombosis or [fatal] PE) during follow-up (equivalent to 1 minus negative predictive value). It is widely appreciated that by consensus the upper bound of the 95% confidence interval of this safety proportion should not exceed 3%. Efficiency is defined as the proportion of patients in whom PE is ruled out based on a low CDR score and normal D-dimer levels (equivalent to the false negatives and true negatives, relative to all patients). Both efficiency and safety will be calculated for each CDR in each clinical setting as well. Between-study heterogeneity and clustering of data in our IPD set will be analyzed using appropriate statistical techniques using a two-stage approach, i.e., first estimate the respective diagnostic indices within each study and then meta-analyze these indices conventionally, using a bivariate random-effects approach. This bivariate approach incorporates any correlation between pairs of (logit transformed) sensitivity and specificity, or predictive values, from the studies in a random-effects meta-analysis [9, 10, 18].

#### Research domain 2: is the predictive performance of each CDR different in various clinically important subgroups?

We define the following clinically important subgroups: active cancer (as defined in the original publication), history of previous venous thromboembolism, inpatients, age (50+, 70+, etc.), gender, and comorbidities such as heart failure and/or COPD where available. To assess the impact of these subgroups on the predictive performance of each CDR, a logistic model will be fitted for each CDR, including with and without (dichotomized) results of D-dimer testing in our dataset. Hereto, the original intercept and regression coefficients will be used, or if not available, the total score of the respective CDR. As such, for each patient included in the IPDMA, a predicted probability of PE is estimated using both the predictors from the CDR and thus with and without results from D-dimer testing. Next, we will perform one-stage meta-analysis with a study-wise intercept term (i.e., fixed effects), the logit of the estimated predicted probability (or risk) as an offset term (i.e., no regression coefficient is estimated for term), and the subgroup covariate as a random effect. If the regression coefficient for this subgroup covariate yields a clinically plausible and statistically significant effect (*p* value arbitrarily 0.10 to 0.15), the conclusion will be that the respective CDR is not well calibrated for this subgroup of patients. In this context, further subgroup effects for this group of patients need to be explored, first by changing the logit of predicted risk from an offset term to a random effect term to check if the respective CDR on average calibrates well in our IPDMA (i.e., the mean slope of this covariate should at least approach 1; essentially, this has also been tested under research domain 1). Finally, to further quantify subgroup effects, this model is expanded using interaction terms of our pre-defined subgroups with (logit) of predicted risk (random effect) [10]. With these models, the mean estimated probability of PE will be estimated for each CDR score separately for each subgroup variable. To illustrate potential heterogeneity, a 95% prediction interval (PI) is calculated for these thus estimated PE probabilities. This 95% prediction interval can be seen as the range of possible PE probabilities for each CDR score, plus the presence or absence of the respective subgroup covariate. As such, wide 95% PIs can be considered as an indication of heterogeneity, warranting further exploration of its associated causes. As a first step, we will then repeat the above-described analyses in more homogenous populations, i.e., those described under research domain 1 (i.e., open-access emergency care, primary healthcare, referred hospital care, and institutionalized patients). If this indeed leads to less wide 95% PIs, observed heterogeneity can be explained by differences in baseline risk.

#### Research domain 3: can the efficiency and safety of ruling out PE across a broad spectrum of patients be improved with a new clinical decision model that combines clinical items with quantitative D-dimer testing?

Historically, the development of CDRs has been aimed at the derivation of simple scores, since they are meant to be calculated at the bedside to rapidly determine which patients should be referred for D-dimer testing or imaging. Therefore, most clinical decision rules consist of about six to eight items, which are traditionally assigned rounded points based on the regression coefficient from a multivariate model. For the sake of simplicity, continuous variables are always dichotomized and potential interaction between items is ignored. In addition, the derivation of most scores did not follow the methodological principles that are nowadays recommended, such as the use of (multilevel) multiple imputations, bootstrapping, and shrinkage. Furthermore, D-dimer testing is often not modeled within the underlying logistic model. Contrastingly, a two-step approach is used: if a clinical decision rule result indicates a low probability of PE, negative D-dimer testing is used to select those patients in whom imaging can be safely withheld. In this setting, various D-dimer thresholds have been proposed: the conventional, fixed threshold, an age-adjusted threshold, and a threshold dependent on the clinical pre-test probability. Although these thresholds appear to be safe in excluding PE, they are all used di- or trichotomously after clinical pre-test probability has been assessed, thereby ignoring the full predictive value of the quantitative D-dimer result; it is well known, e.g., that higher D-dimer levels are associated with a higher PE probability.

To overcome the methodological limitations of the present clinical decision rules and improve PE risk prediction, we aim to derive a new clinical decision model consisting of both clinical items as well the quantitative D-dimer result. An IPD dataset provides an excellent framework for this purpose. The increasing use of smartphone applications and websites for the calculation of risk scores provides several advantages: continuous variables can be used without dichotomization, interactions between variables can be assessed, and risk prediction can be tailored to the healthcare setting or known disease prevalence. Moreover, it will be possible to provide an absolute, individualized PE probability rather than a probability range.

Hereto, for such a full clinical decision model, various well-known risk factors for PE, signs and symptoms of PE, and the quantitative D-dimer result will be considered in an overall multilevel, multivariable logistic regression model. Continuous variables will be transformed if appropriate and clinically plausible interactions will be explored (e.g., active cancer with age, D-dimer with age, and D-dimer with gender). Variables in the final model will be selected using stepwise backward selection. Bootstrapping techniques will be used to internally validate the model and shrink coefficients accordingly (if needed). Diagnostic performance of this updated CDR will be evaluated using traditional statistical approaches, similar as those described with research domain 1 (c-index, calibration, diagnostic indices). Finally, we intend to internally validate the new model separately in each of the existing datasets using internal-external cross-validation techniques [19]. With this technique, the new model will be derived in the total IPDMA set while iteratively excluding one dataset in which the model is subsequently validated. Thus, multiple derivation models are fit and next validated. Next, model performance is explored in each validation set separately by assessing both discrimination of the (respective) derived model (c-statistic) and calibration (expected versus observed ratio, and—graphically—the calibration slope in a calibration plot). Ideally, all thus performed model validations should perform well in each validation set, thereby providing proof that the full IPDMA set can be used in total for model derivation. Should scenarios unfold where model validation is poor in one or more validation samples, this implies that generalizability of the derived model cannot be guaranteed across all patient populations, either due to heterogeneity in baseline risk (i.e., the intercept of the model) or heterogeneity across predictor-outcome associations (or both). In this situation, we will (clinically and statistically) explore model derivation in more homogenous datasets as included in our IPDMA and subsequently explain to what patient populations the thus derived new model may be (or may not be) applicable and suitable for subsequent validation studies in newly derived prospective datasets in new studies.

### Risk of bias assessment

No formal risk of bias assessment tool currently exists for scoring the risk of bias in prediction model studies. However, at recent meetings of the Cochrane Collaboration, the so-called PROBAST tool (Prediction model study Risk Of Bias ASsessment Tool) is presented, but is not yet formally published. The CHARMS guideline that is developed for framing the review question for systematic reviews of prediction model studies, and for guiding the data extraction and critical appraisal of primary prediction model studies, provides guidance on risk of bias in these particular studies as well [20]. As such, we will use the CHARMS guideline in combination with preliminary version of the PROBAST tool to construct a checklist for the risk of bias assessment of the selected studies for this IPDMA.

## Discussion

Pulmonary embolism is a major healthcare burden and remains a diagnostic challenge given its often non-specific clinical presentation and varying performance of the currently recommended diagnostic strategies across different healthcare settings, patient characteristics, and comorbidity. Physicians have since long been struggling with this clinical conundrum. This IPDMA will address these issues and aims at diagnostic assessment tailored to different health care settings and to individual patients. Currently, we are in the final phase of building our dataset with a dedicated group of expert investigators worldwide. We expect to publish our first results late 2018 or early 2019.

## Appendix

### Search strategy

((Validat$ OR Predict$.ti. OR Rule$) OR (Predict$ AND (Outcome$ OR Risk$ OR Model$)) OR ((History OR Variable$ OR Criteria OR Scor$ OR Characteristic$ OR Finding$ OR Factor$) AND (Predict$ OR Model$ OR Decision$ OR Identif$ OR Prognos$)) OR (Decision$ AND (Model$ OR Clinical$ OR Logistic Models/)) OR (Prognostic AND (History OR Variable$ OR Criteria OR Scor$ OR Characteristic$ OR Finding$ OR Factor$ OR Model$)) OR (“Stratification” OR “ROC Curve”[Mesh] OR “Discrimination” OR “Discriminate” OR “c-statistic” OR “c statistic” OR “Area under the curve” OR “AUC” OR “Calibration” OR “Indices” OR “Algorithm” OR “Multivariable”))

AND

(“pulmonary embolism”[MeSH Terms] OR (“pulmonary”[All Fields] AND “embolism”[All Fields]) OR “pulmonary embolism”[All Fields])

## Abbreviations

CDR: Clinical decision rule
CTPA: Computed tomographic pulmonary angiography
DVT: Deep vein thrombosis
IPDMA: Individual patient data meta-analysis
PE: pulmonary embolism

## References

[1] Le Gal G, Bounameaux H (2004). Diagnosing pulmonary embolism: running after the decreasing prevalence of cases among suspected patients. J Thromb Haemost.

[2] Prasad V, Rho J, Cifu A (2012). The diagnosis and treatment of pulmonary embolism: a metaphor for medicine in the evidence-based medicine era. Arch Intern Med.

[3] Carrier M, Kimpton M, Le Gal G, Kahn SR, Kovacs MJ, Wells PS (2011). The management of a sub-segmental pulmonary embolism: a cross-sectional survey of Canadian thrombosis physicians. J Thromb Haemost.

[4] Lucassen W, Geersing GJ, Erkens PMG, Reitsma JB, Moons KGM, Büller H (2011). Clinical decision rules for excluding pulmonary embolism: a meta-analysis. Ann Intern Med.

[5] Geersing GJ, De Groot JA, Reitsma JB, Hoes AW, Rutten FH (2015). The impending epidemic of chronic cardiopulmonary disease and multimorbidity: the need for new research approaches to guide daily practice. Chest.

[6] Van Es N, Van Der Hulle T, Van Es J, Den Exter PL, Douma RA, Goekoop RJ (2016). Wells rule and d-dimer testing to rule out pulmonary embolism a systematic review and individual-patient data meta-analysis. Ann Intern Med.

[7] van der Hulle T, den Exter PL, Mos ICM, Kamphuisen PW, Hovens MMC, Kruip MJHA (2014). Optimization of the diagnostic management of clinically suspected pulmonary embolism in hospitalized patients. Br J Haematol.

[8] Schouten HJ, Geersing GJ, Koek HL, Zuithoff NP, Janssen KJ, Douma RA (2013). Diagnostic accuracy of conventional or age adjusted D-dimer cut-off values in older patients with suspected venous thromboembolism: systematic review and meta-analysis. BMJ.

[9] Debray TPA, Riley RD, Rovers MM, Reitsma JB, Moons KGM (2015). Individual participant data (IPD) meta-analyses of diagnostic and prognostic modeling studies: guidance on their use. PLoS Med.

[10] Ahmed I, Debray TP, Moons KG, Riley RD (2014). Developing and validating risk prediction models in an individual participant data meta-analysis. BMC Med Res Methodol.

[11] Geersing GJ, Zuithoff NPA, Kearon C, Anderson DR, Ten Cate-Hoek AJ, Elf JL, et al. Exclusion of deep vein thrombosis using the Wells rule in clinically important subgroups: individual patient data meta-analysis vein thrombosis using the Wells rule in clinically important subgroups: individual patient data meta-analysis. BMJ. 2014;348:g1340. 10.1136/bmj.g1340 PMC3948465 24615063

[12] Stewart LA, Clarke M, Rovers M, Riley RD, Simmonds M, Stewart G (2015). Preferred reporting items for a systematic review and meta-analysis of individual participant data: the PRISMA-IPD statement. JAMA.

[13] Shamseer L, Moher D, Clarke M, Ghersi D, Liberati A, Petticrew M (2015). Preferred reporting items for systematic review and meta-analysis protocols (PRISMA-P) 2015: elaboration and explanation. BMJ.

[14] Geersing GJ, Bouwmeester W, Zuithoff P, Spijker R, Leeflang M, Moons K. Search filters for finding prognostic and diagnostic prediction studies in MEDLINE to enhance systematic reviews. PLoS One. 2012;7(2):e32844. 10.1371/journal.pone.0032844 PMC3290602 22393453

[15] Jolani S, Debray TPA, Koffijberg H, van Buuren S, Moons KGM (2015). Imputation of systematically missing predictors in an individual participant data meta-analysis: a generalized approach using MICE. Stat Med.

[16] Hendriksen JMT, Geersing G-J, Lucassen WAM, Erkens PMG, Stoffers HEJH, van Weert HCPM (2015). Diagnostic prediction models for suspected pulmonary embolism: systematic review and independent external validation in primary care. BMJ.

[17] van der Hulle T, Cheung WY, Kooij S, Beenen LFM, van Bemmel T, van Es J, et al. Simplified diagnostic management of suspected pulmonary embolism (the YEARS study): a prospective, multicentre, cohort study. Lancet. 2017;6736 10.1016/S0140-6736(17)30885-1. 10.1016/S0140-6736(17)30885-1 28549662

[18] Riley RD, Ensor J, Snell KIE, Debray TPA, Altman DG, Moons KGM (2016). External validation of clinical prediction models using big datasets from e-health records or IPD meta-analysis: opportunities and challenges. BMJ.

[19] Debray TPA, Moons KGM, Ahmed I, Koffijberg H, Riley RD (2013). A framework for developing, implementing, and evaluating clinical prediction models in an individual participant data meta-analysis. Stat Med.

[20] Moons KGM, de Groot JAH, Bouwmeester W, Vergouwe Y, Mallett S, Altman DG, et al. Critical appraisal and data extraction for systematic reviews of prediction modelling studies: the CHARMS Checklist. PLoS Med. 2014;11(10):e1001744. 10.1371/journal.pmed.1001744 PMC4196729 25314315

[21] Wells PS, Anderson DR, Rodger M, Ginsberg JS, Kearon C, Gent M (2000). Derivation of a simple clinical model to categorize patients probability of pulmonary embolism: increasing the models utility with the SimpliRED D-dimer. Thromb Haemost.

[22] Le Gal G, Righini M, Roy PM, Sanchez O, Aujesky D, Bounameaux H (2006). Prediction of pulmonary embolism in the emergency department: the revised Geneva score. Ann Intern Med.

[23] Kline JA, Mitchell AM, Kabrhel C, Richman PB, Courtney DM (2004). Clinical criteria to prevent unnecessary diagnostic testing in emergency department patients with suspected pulmonary embolism. J Thromb Haemost.

[24] van der Hulle T, Cheung WY, Kooij S, Beenen LFM, van Bemmel T, van Es J (2017). Simplified diagnostic management of suspected pulmonary embolism (the YEARS study): a prospective, multicentre, cohort study. Lancet.

